# Rescuing macrophage normal function in spinal cord injury with embryonic stem cell conditioned media

**DOI:** 10.1186/s13041-016-0233-3

**Published:** 2016-05-06

**Authors:** Lei Guo, Alyssa J. Rolfe, Xi Wang, Wenjiao Tai, Zhijian Cheng, Kai Cao, Xiaoming Chen, Yunsheng Xu, Dongming Sun, Jinhua Li, Xijing He, Wise Young, Jianqing Fan, Yi Ren

**Affiliations:** Department of Orthopedics, The Second Affiliated Hospital of Xian Jiaotong University, Xian, 710004 China; Department of Biomedical Sciences, Florida State University, College of Medicine, 1115 West Street, Tallahassee, FL 32306 USA; W. M. Keck Center for Collaborative Neuroscience, Rutgers, The State University of New Jersey, New Brunswick, NJ 08854 USA; Institute of Inflammation and Diseases, the First Affiliated Hospital of Wenzhou Medical University, Wenzhou, China; Department of Anatomy and Developmental Biology, Monash University, Clayton, VIC 3800 Australia; Statistical Laboratory, Princeton University, Princeton, NJ 08540 USA

**Keywords:** Embryonic Stem Cells (ESCs), Spinal Cord Injury (SCI), Myelin, Bone Marrow-Derived Macrophages (BMDMs), Microglia, Inflammation

## Abstract

**Background:**

Macrophages play an important role in the inflammatory responses involved with spinal cord injury (SCI). We have previously demonstrated that infiltrated bone marrow-derived macrophages (BMDMs) engulf myelin debris, forming myelin-laden macrophages (mye-Mϕ). These mye-Mϕ promote disease progression through their pro-inflammatory phenotype, enhanced neurotoxicity, and impaired phagocytic capacity for apoptotic cells. We thus hypothesize that the excessive accumulation of mye-Mϕ is the root of secondary injury, and that targeting mye-Mϕ represents an efficient strategy to improve the local inflammatory microenvironment in injured spinal cords and to further motor neuron function recovery. In this study, we administer murine embryonic stem cell conditioned media (ESC-M) as a cell-free stem cell based therapy to treat a mouse model of SCI.

**Results:**

We showed that BMDMs, but not microglial cells, engulf myelin debris generated at the injury site. Phagocytosis of myelin debris leads to the formation of mye-Mϕ in the injured spinal cord, which are surrounded by activated microglia cells. These mye-Mϕ are pro-inflammatory and lose the normal macrophage phagocytic capacity for apoptotic cells. We therefore focus on how to trigger lipid efflux from mye-Mϕ and thus restore their function. Using ESC-M as an immune modulating treatment for inflammatory damage after SCI, we rescued mye-Mϕ function and improved functional locomotor recovery. ESC-M treatment on mye-Mϕ resulted in improved exocytosis of internalized lipids and a normal capacity for apoptotic cell phagocytosis. Furthermore, when ESC-M was administered intraperitoneally after SCI, animals exhibited significant improvements in locomotor recovery. Examination of spinal cords of the ESC-M treated mice revealed similar improvements in macrophage function as well as a shift towards a more anti-inflammatory environment at the lesion and parenchyma.

**Conclusions:**

The embryonic stem cell conditioned media can be used as an effective treatment for SCI to resolve inflammation and improve functional recovery while circumventing the complications involved in whole cell transplantation.

## Background

Spinal cord injury (SCI) results in a debilitating chronic condition, complicated by costly and complex rehabilitation challenges [1]. The pathophysiology of an acute SCI comprises both primary and secondary mechanisms of injury [2]. The primary injury, resulting from mechanical trauma to the spinal cord, leads to immediate vasospasm of the superficial vessels and intraparenchymal hemorrhage [3]. The culmination of the events of the acute primary injury stage initiates the secondary injury cascade. The secondary injury is characterized by a prolonged inflammatory response [4, 5], resulting in further tissue damage and neurodegeneration by resident microglia and infiltrated bone marrow-derived macrophages (BMDMs) [6].

In the spinal cord, the clearance of apoptotic cells and myelin debris is the responsibility of infiltrating macrophages, and to a lesser degree, resident microglia. The myelin debris contains a variety of myelin-associated inhibitors of axonal regeneration including Nogo-A, myelin-associated glycoprotein (MAG) and myelin-oligodendrocyte glycoprotein (MOG) [7]. Additionally, large numbers of circulating neutrophils infiltrate in the first day after injury [8] where they are frequently present in the injury site for more than 40 days [9]. If apoptotic neutrophils can’t be efficiently cleared by macrophages from injury site, these uningested apoptotic neutrophils undergo secondary necrosis and it is inevitable that there is an uncontrolled release of toxic intracellular contents from necrotic neutrophils into the tissue where it causes further damage [10]. Thus, prompt clearance of the array of apoptotic cells and myelin debris by macrophages is critical to limit the inflammatory cascade and promote neuroregeneration [8].

Along with the cell debris generated following SCI, there is also dramatic accumulation of lipids derived from myelin debris in the lesion extending far beyond the epicenter [11, 12]. Persistence of these lipids is part of the chronic pathology and results in lipid peroxidation, one of the most damaging side-effects of lipid retention. Lipid homeostasis in the injured spinal cord is highly dysregulated, leading to the formation of persistent foamy lipid laden macrophages. Our previous studies have demonstrated the pro-inflammatory characteristics of these foamy macrophages, which include but are not limited to enhanced neurotoxicity, and impaired wound healing [8]. Here we have shown that dysregulation of macrophage lipid homeostasis in the injured cord, is the major contributing factor of long term lipid retention. We propose that these pro-inflammatory myelin-laden macrophages (mye-Mϕ) are an important therapeutic target in SCI because of their reduced ability to clear apoptotic cells and myelin debris at the injury site.

Embryonic stem cells (ESCs) have shown therapeutic potential to remyelinate axons as ESC derived oligodendrocyte precursor cells (OPCs) can generate functional myelin in situ [13]. Their application in SCI though may have limited success due to the risk of tumor formation after transplantation and immune rejection [14, 15]. Our previous studies have shown that ESCs produce factors can induce anti-inflammatory phenotype in macrophages [16, 17]. Upon ESC stimulation, phagocytic function was enhanced and macrophages expressed multiple angiogenic growth factors and proteinases promoting angiogenesis [16, 17]. These properties correspond with the characteristics required for successful treatment for SCI pathology, driving us to investigate the novel application of ESC derived factors for treating inflammation in acute murine SCI.

## Results

### Myelin-laden macrophages in vivo and in vitro

**Fig. 1:**
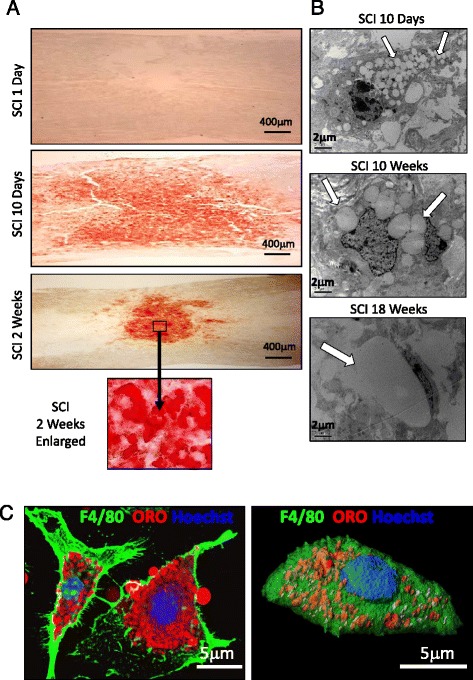
Mye-Mϕ formation in vivo and in vitro. **a** Using Oil Red O (ORO) staining, lipid accumulation at the injury site can be observed starting as diffuse staining at 10 days and by 2 weeks after SCI. There is a central core of high intensity staining at 2 weeks post SCI. **b** Electron micrographs of macrophages in the injured spinal cord at indicated time points. **c** In vitro induction of mye-Mϕ. BMDMs (F4/80^+^) incubated with myelin debris and intracellular lipids were labeled by ORO. 3D reconstruction by confocal microscopy shows the myelin-derived lipids distributed throughout the cell subsequent to myelin treatment (right)

Myelin debris persists for extended periods of time in the injured spinal cord [18, 19], where it is retained intracellularly by macrophages. We have previously demonstrated that infiltrated bone marrow-derived macrophages (BMDMs) migrate to the epicenter of the injured spinal cord. In the injury site, they phagocytose myelin debris leading to the formation of myelin-laden macrophages (mye-Mϕ) which are distinguished as Oil Red O (ORO) positive cells [8]. These mye-Mϕ take on a foamy appearance due to the large number of intracellular lipid droplets, and are a hallmark of phagocytic activity in atherosclerosis [20] and demyelinating diseases such as experimental allergic encephalomyelitis (EAE) and multiple sclerosis (MS) [21]. ORO selectively stains intracellular neutral lipids such as cholesterol ester (CE) but not intact myelin in the normal central nervous system (CNS) and peripheral nervous system (PNS). ORO-positive lesions reveal foamy cell accumulation in the injury site. Figure 1a shows high intensity ORO staining localized to the lesion epicenter at 2 weeks post-SCI. Using electron microscopy, we find lipid droplets (arrows) in the cytoplasm of the mye-Mϕ at the injury site. At 10 days post-SCI, the small evenly distributed morphology of the droplets is comparable to those observed in vitro. At 10 weeks, there is a noticeable decrease in the number of intracellular lipid droplets as well as an increase in the size per droplet. The trend towards fewer and larger droplets continues up to 18 weeks post-SCI, at which point a single droplet per cell can be observed (Fig. 1b). The same lipid accumulation is observed in vitro when BMDMs are incubated in the presence of myelin debris as demonstrated by F4/80^+^ mye-Mϕ with small lipid droplets distributed throughout the cytoplasm (Fig. 1c).

### BMDMs are the primary myelin phagocytosing cells in SCI

**Fig. 2:**
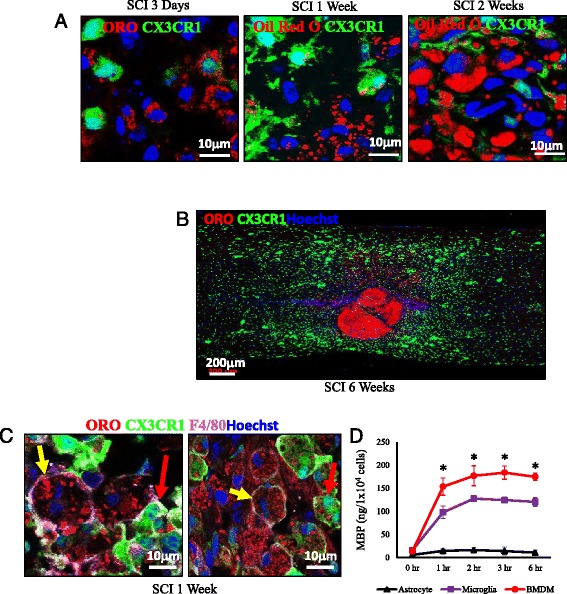
Macrophages are the Primary Myelin Phagocytosing Cells in SCI. Using a CX3CR1^GFP/+^ transgenic mouse model of SCI, tissue sections were stained with ORO to show the localization of lipids within cells after injury. **a** The distribution of lipid droplets in CX3CR1 strongly positive cells (microglia, CX3CR1^+++^) and weakly positive or negative cells (BMDMs, CX3CR1^-/±^) at the lesion sites at different time points after SCI. **b** Imaging of the entire injury site at 6 weeks shows the progressive exclusion of microglia (CX3CR1^+++^) from epicenter where the majority of the lipids are retained. **c** Staining of F4/80 provides clear cell membrane boundaries to further validate the localization of the lipids predominantly within the BMDMs (F4/80^+^CX3CR1^-/±^, yellow arrows) with less being localized to the microglia (F4/80^+^CX3CR1^+++^, red arrows). **d** Quantification of myelin debris phagocytosis in BMDMs, astrocyte, and microglia by detecting intracellular MBP by ELISA (*n* = 3). (*p < 0.05) Data are represented as means ± SD

As both macrophages and their nervous system counterparts, microglia, are considered professional phagocytes [22], we sought to address their individual contribution in the lipid laden at injury epicenter. We implemented a CX3CR1^GFP/+^ transgenic mouse in which all microglial cells express strongly GFP, while BMDMs are weakly positive [8]. As early as 3 days, ORO staining revealed intracellular lipid accumulation at the injury site. However, little to no lipid staining was detected within CX3CR1^+^ cells, indicating that myelin phagocytosis was carried out almost entirely by infiltrated BMDMs, not by microglial cells (Fig. 2a). This pattern of lipid localization is observed at 1 week and 2 weeks, with an increasing lack of ORO signal co-localization in the microglia (Fig. 2a). Six weeks post SCI, microglia are excluded from the injury epicenter where the high intensity ORO staining is present along with BMDMs (Fig. 2b). In order to confirm the BMDMs are the major myelin debris scavenger cells in SCI, tissue sections were stained with F4/80, a membrane protein expressed on macrophages and microglia. BMDMs are F4/80^+++^/CX3CR1^−/±^, while microglia are F4/80^+++^/CX3CR1^+++^. This aided to further clarify that the lipids are intracellular and co-localize with BMDMs (yellow arrows) more frequently than microglia (red arrows) (Fig. 2c). While a major function of both BMDMs and microglia is phagocytosis, astrocytes are also known to have a limited capacity to clear debris in neurodegenerative disease states [22]. To further validate that BMDMs are the primary cells responsible for engulfing myelin debris, quantification of the myelin phagocytic capacity of BMDMs, microglia, and astrocytes was performed. Myelin debris was incubated with primary cultures of the three of cell types for the indicated time, at which point the intracellular myelin basic protein (MBP) was determined. Microglial cells showed a moderate ability to engulf myelin but it was significantly less than BMDMs. Unsurprisingly, the contribution of the astrocytes to the myelin debris phagocytosis was exceedingly low (Fig. 2d).

### Consequences of impaired mye-Mϕ phagocytosis in the injured spinal cord

**Fig. 3:**
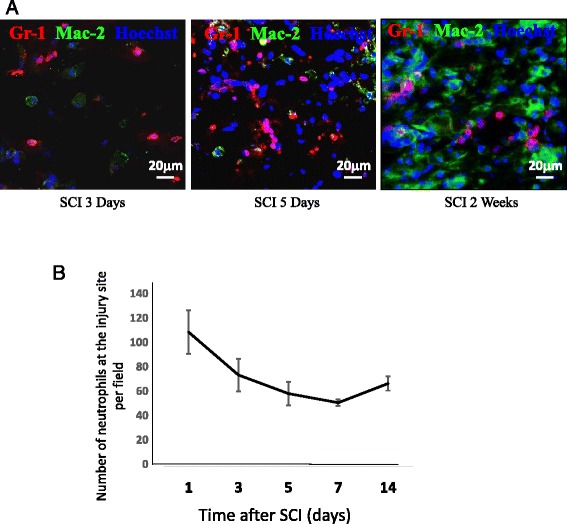
Neutrophil accumulation at the injured spinal cord. **a** Immunohistochemical analysis showing neutrophils (Gr-1^+^, red) at the lesion site at indicated time after SCI. Infiltrated BMDMs were labeled by Mac-2 antibody (green). **b** Quantification of neutrophil infiltration at the injury site (*n* = 3). Data are represented as means ± SD

Neutrophils play an important role in inflammation initiation and resolution. In the case of non-resolving inflammation, neutrophils persist at the inflamed site where they can cause additional tissue destruction [23, 24]. Moreover, the removal of neutrophils and their potentially histotoxic contents is a prerequisite of inflammation resolution [25, 26]. Given that our previous in vitro data revealed mye-Mϕ have a reduced efferocytosis capacity [8], we hypothesized that there is a prolonged accumulation of neutrophils in injured spinal cord from mye-Mϕs failing to clear them. At 5 days post-SCI, neutrophils can still be observed in the injury site along with BMDMs (Mac-2, the marker for BMDMs [8]). Of the neutrophils present, few co-localized with BMDMs, consistent with a lack of phagocytosis (Fig. 3). Up to 2 weeks post SCI, neutrophils are still observed in the lesion outside of macrophages indicating a lack of clearance.

### ESC-M regulates inflammatory response

**Fig. 4:**
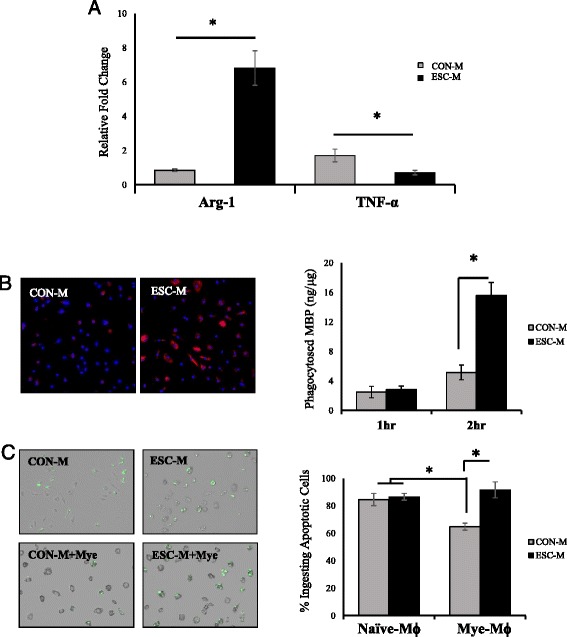
The effects of ESC-M on regulation of macrophage activation and phagocytosis. **a** BMDMs were incubated with myelin debris to induce mye-Mϕ, and then incubated with CON-M or ESC-M for 24 h respectively. The mRNA levels of TNF-α and arginase-1 (Arg-1) were determined by RT-PCR (*n* = 4). Fold change values were normalized to mye-Mϕ treated with CON-M. **b** BMDMs were pre-treated with CON-M or ESC-M for 24 h and then incubated with myelin debris for 1 or 2 h. The myelin lipids engulfed were stained by ORO (left, original magnification, ×200). Intracellular MBP was assayed by ELISA to determine the quantity of phagocytosed myelin debris (right, *n* = 3). **c** Naïve-Mϕ (BMDMs without myelin treatment) or mye-Mϕ were treated with CON-M or ESC-M for 24 h and then incubated with apoptotic thymocytes labeled with CFSE for 30 min to test apoptotic cell phagocytosis (left, original magnification, ×200). The percentage of ingested apoptotic cells were calculated (right, *n* = 4). (*p < 0.05) Data are represented as means ± SD

We previously demonstrated myelin debris is a potent pro-inflammatory stimulus that inhibits M2-like macrophage activation and enhances expression of pro-inflammatory cytokines such as tumor necrosis factor-α (TNF-α) [8, 12]. We also showed that ESCs are able to polarize BMDMs into M2-like macrophages [16, 17], thus we reasoned that ESCs may have anti-inflammatory properties. To explore the potentially anti-inflammatory effects of ESCs on mye-Mϕ, we used ESC conditioned media (ESC-M) as a cell-free treatment. BMDMs were cultured with myelin debris for 24 h to induce mye-Mϕ and then treated with CON-M or ESC-M for 24 h prior to lysis and RNA extraction. ESC-M treatment significantly reduced TNF-α expression in mye-Mϕ compared to CON-M treated mye-Mϕ (Fig. 4a). Not only did ESC-M decrease pro-inflammatory TNF-α transcription, it also significantly increased expression of arginase-1 (Arg-1), the well-documented M2 marker, compared to CON-M treated mye-Mϕ (Fig. 4a). These data suggests that ESCs produce factors which may directly augment the inflammatory response by regulating myelin-induced macrophage activation.

### ESC-M enhances myelin debris clearance and rescues impaired mye-Mϕ phagocytic capacity for apoptotic cells

Myelin debris removal and apoptotic cell clearance are critical for axon remyelination and resolution of inflammation, making them important therapeutic targets. To test if ESC-M would be useful for this application as well, BMDMs were treated with CON-M and ESC-M for 24 h then incubated with myelin debris for 1 or 2 h. Visually, ORO staining revealed large amounts of intracellular lipids associated with each cell in the ESC-M treated group indicating high levels of phagocytosis (Fig. 4b). MBP ELISA confirmed ESC-M treated cells contained significantly higher amounts of intracellular MBP than CON-M at 2 h which is correlated to increased phagocytosis of myelin debris (Fig. 4b). To assess the phagocytosis of apoptotic cells in response to ESC-M, BMDMs were incubated with myelin debris for 24 h to induce mye-Mϕ, then incubated in ESC-M or CON-M for an additional 24 h. Apoptotic cells labeled with CFSE were then added to macrophages at a concentration of 10:1 for 30 min. Non-phagocytosed cells were washed away and macrophages containing CFSE positive cells were quantified (Fig. 4c). The phagocytosis of apoptotic cells in mye-Mϕ is significantly inhibited but, ESC-M treatment rescued the impaired phagocytosis of the mye-Mϕ (Fig 4c). These results represent a novel function for ESC secreted factors to enhance the phagocytosis of myelin debris and rescue the mye-Mϕ clearance of apoptotic cells.

### ESC-M enhances lipid efflux from mye-Mϕ reducing the formation of foamy cells

**Fig. 5:**
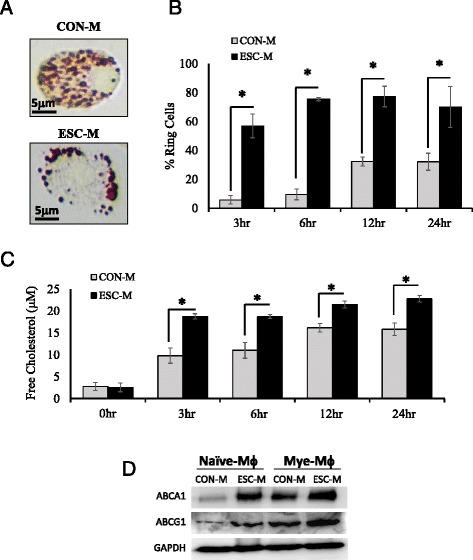
ESC-M Enhances Lipid Efflux from Mye-Mϕ Reducing the Formation of Foamy Cells. **a** BMDMs exocytosing lipids can be visualized with a ring of lipid droplets (ring cells) on the cell periphery. **b** Mye-Mϕ were treated with CON-M and ESC-M, respectively for indicated time points. The number of foam cells exocytosing lipids was quantified as the percentage of total cells with ring cells (*n* = 5). **c** The product of reverse cholesterol transport is free extracellular cholesterol which was quantified with a flourometric assay (*n* = 4). **d** Naïve-Mϕ and mye-Mϕ were incubated with CON-M and ESC-M for 24 h. The expression of ABCA1 and ABCG1 were determined by Western Blot. (*p < 0.05) Data are represented as means ± SD

Because the ESC-M treated mye-Mϕ showed increased myelin debris engulfment and normal apoptotic cell phagocytosis, we reasoned that this was related to the rescued efflux capacity of intracellular lipids. To test if ESC-M is also able to increase reverse cholesterol transport, BMDMs were loaded with myelin debris for 2 h and undigested myelin debris was washed away. Staining with ORO revealed neutral lipid droplets localized to the periphery of the cell membrane in the ESC-M treated cells and a diffuse pattern in CON-M cells. The localization of lipids to the periphery is a prerequisite for efflux (Fig 5a). This staining pattern was defined ring cells and the percentage of cells that exhibited this arrangement was quantified. At all time points, the ESC-M enhanced lipid droplet trafficking to the cell periphery (Fig. 5b). To confirm that the peripheral lipids in the ring cells were in fact exocytosed lipids, free cholesterol in the culture media was assayed. Once again, at all time points the ESC-M showed enhanced cholesterol efflux as measured by free cholesterol concentrations (Fig. 5c). The major cholesterol efflux transporters in macrophages are the ATP-binding cassette family members, ABCA1 and ABCG1 [27]. Western blotting for these transporters in macrophages indicated that ESC-M enhances the expression of both transporters compared to CON-M. In both media treatments, the addition of myelin debris increased transporter expression as expected, although the ESC-M with myelin treatment showed still a larger increase than CON-M with myelin (Fig 5d). This observation represents a novel function of ESCs to trigger lipid efflux from foam cells.

### ESC-M improves locomotor recovery

**Fig. 6:**
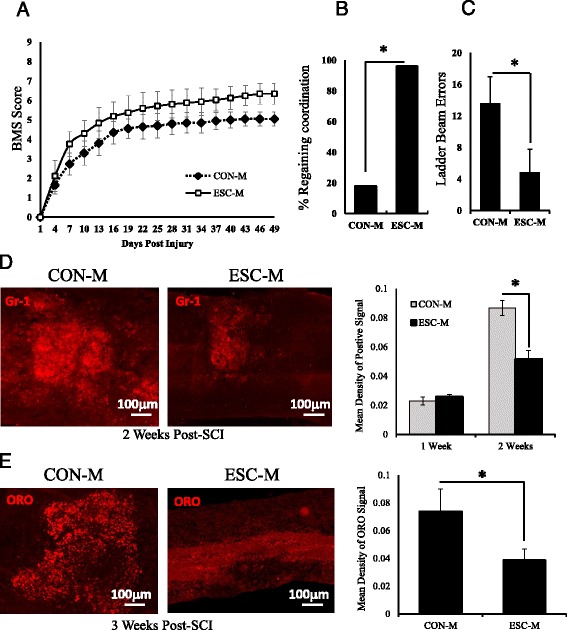
ESC-M Reduces Lipid and Neutrophil Accumulation and Improves Locomotor Recovery. SCI mice were injected (*i.p.*) with ESC-M (*n* = 25) or CON-M (*n* = 17) (1 mL per mouse) after surgery and every 3^rd^ subsequent day. **a** The locomotor function was assessed by Basso Mouse Scale (BMS) (ANOVA, *p* < 0.05). **b** Recovery of coordination in CON-M and ESC-M treated mice using chi square analysis for observed frequency. **c** The alternative horizontal ladder beam coordination based measure of locomotor activity on mice treated with CON-M or ESC-M. **d** Neutrophil accumulation was determined by detecting Gr-1^+^ cells in SCI mice treated with CON-M and ESC-M for 1 and 2 weeks (*n* = 4). **e** Lipid accumulation was determined by ORO staining in SCI mice treated with CON-M and ESC-M for 3 weeks (*n* = 4). (*p < 0.05) Data are represented as means ± SD

To test whether improved macrophage function could be enhanced in an acute SCI model, one mL of ESC-M or CON-M was injected intraperitoneally (*i.p.*) immediately after the surgery and every 3 subsequent days. To assess the ability of this treatment method to improve functional outcomes, we implemented three measures of locomotor activity, the BMS scale [28], regained coordination [29], and the horizontal ladder beam test [30]. Comparing to CON-M group, BMS scores of the ESC-M group were significantly higher (ANOVA, *p* <0.05) after the first day. At 7 weeks post-injury (termination of the study) the average BMS scores per group were: ESC-M (*n* = 25) 6.3, CON-M (*n* = 17) 5.1 (Fig. 6a). These scores corresponded to the improvement in the frequency of coordination test for ESC-M vs. CON-M groups. We used Chi-squared frequency analysis to compare the percentage of mice in each group that had regained at least occasional coordination in the locomotor assessment. Recovery of coordination was significantly increased in ESC-M group (96 %) compared to CON-M group (20 %) (Fig. 6b). Additionally, the horizontal ladder beam task suggested that ESC-M treated mice exhibited significantly fewer errors than CON-M treated mice (Fig. 6c).

### ESC-M reduces lipid accumulation and regulates the inflammatory response in the injured spinal cord

As in vitro phagocytosis assays had already shown rescued phagocytic capacity following ESC-M treatment, we sought to assess this effect in vivo. Staining for Gr-1 was performed to determine the ability of ESC-M treated animals to clear neutrophils, a critical step in termination of the inflammatory cascade [10]. After one week, no difference in neutrophil accumulation is present, but at two weeks post-SCI fewer neutrophils are present in the injury site of ESC-M treated group compared to CON-M treated group (Fig. 6d). We also investigated lipid accumulation in the injury because our in vitro results had demonstrated improved resolution of foamy cells through enhanced ABCA1 expression following ESC-M treatment. Using ORO staining, at 3 weeks post-SCI the accumulated lipid area of ESC-M group was significantly less than CON-M group (Fig 6e). This suggests that the effects ESC-M has in vitro on rescued phagocytosis and lipid efflux are also present in vivo and correlated to improved functional recovery.

**Fig. 7:**
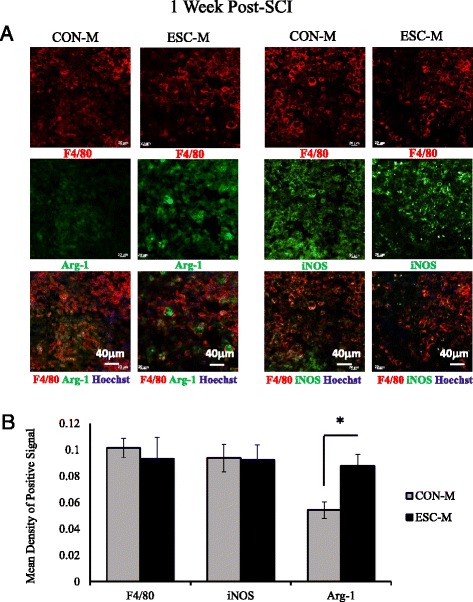
In vivo anti-inflammatory response elicited by ESC-M. **a** Representative images of spinal sections 1 week after injury with double-staining for F4/80/Agr-1 or F4/80/iNOS in CON-M and ESC-M treated mice. **b** The Arg-1 and iNOS expression were quantified by Image Pro Plus 6 (*n* = 3). (*p < 0.05) Data are represented as means ± SD

To determine if the improved recovery correlated with decreased inflammation in the injured spinal cord, we examined the expression of several macrophage activation markers (Fig. 7a). Although ESC-M treatment did not decrease iNOS expression at the injury site, it did significantly increase Arg-1 expression with no change in the number of macrophages at the injury site between groups at 1 week post-SCI (Fig. 7a, b). Because mye-Mϕ have M1-like phenotype [8], our current results suggest that ESC-M treatment may be able to promote the transition of mye-Mϕ from a M1-like to M2-like polarization. Furthermore, it is important to note that the ESC-M treatment does not alter macrophage recruitment to the injury as their role in debris clearance seems critical in promoting functional recovery.

## Discussion

Embryonic stem cell therapies have shown promising clinical applications for various conditions, but they are not without serious ethical and technical limitations. One such technical hurdle that must be overcome is mitigating the risk of teratoma formation associated with ESC derived grafts. To address the possibility of teratoma development, various risk reducing strategies have been developed such as prolonged pre-differentiation in vitro, blocking of proliferative signaling pathways, inducing proliferative ESC apoptosis, sorting cells expressing precursor markers, and deleting undifferentiated ESCs [31–42]. However, it should be noted that it is challenging to obtain a pure culture for safe transplantation, and the contamination of grafts with undifferentiated cells dramatically increases the likelihood of teratoma formation [14, 15, 43]. The method of ESC-M treatment presented here represents a novel therapy which circumvents many of the traditional limitations of ESCs based treatments. Systemic delivery of the treatment can be performed with no observed side-effects and the risk for immune rejection and tumor formation are eliminated. Additionally, application of the ESC secretome independent of whole cells reduces the need for embryonic tissue collection and the ethical concerns associated with obtaining it. In our previous study, we showed that ESCs secrete a combination of molecules, including molecules that recruit macrophages, such as VEGF, MMP-9 and MCP-1 [17]. Also secreted by ESCs are molecules that promote macrophage survival and polarize macrophages towards a M2-like phenotype, such as IL-34 [16]. Moreover, it is also not unreasonable to suggest that if the specific secreted factors regulating recovery can be identified, human therapies could be developed which do not depend on the direct culture of ESCs.

In the present study, we have established a novel application for ESC conditioned media to rescue mye-Mϕ function and promote recovery in a murine SCI model. The ESC-M induced effects can be best characterized as M2-like due to increased Arg-1 expressed in the lesion concurrent with other signs of injury resolution like reduced lipid and neutrophil accumulation. ESC-M treatment can also be considered inflammation reducing as it decreased the expression of TNF-α, a pleotropic cytokine produced by monocytic linage cells. This is of particular significance because TNF-α is considered a master-regulator in chronic inflammatory conditions [44] and is a known regulator of macrophage apoptotic cell efferocytosis [45]. It has also been demonstrated that TNF-α prevents mye-Mϕ conversion from M1-like to M2-like macrophages in vitro and in vivo [46]. The reduced TNF-α therefor represents a possible mechanism for the observed increase in Arg-1, as well as improved cell debris clearance in ESC-M treated animals.

Our previous studies have demonstrated the pro-inflammatory nature of mye-Mϕ and their deleterious role in SCI [8]. Here we also examined the role of resident microglia as they are from the same monocytic lineage and are responsive to similar stimuli. Three independent microscopic methods indicate the intracellular lipids are predominantly localized with BMDMs, despite ability of microglia to likewise phagocytose cell debris. Further quantification of intracellular MBP supported the establishment of BMDMs as the primary myelin phagocytosing cell type with microglia and astrocytes having a lesser role. Because microglia have a weaker phagocytic capacity for myelin debris and are progressively excluded from the epicenter over time, our conclusion that BMDMs are the primary foamy cells was further supported.

The persistence of foamy mye-Mϕ in the injured cord, we reasoned, also had the potential to be mitigated by ESC-M treatment. Macrophages normally efflux intracellular lipids to cholesterol poor proteins in the blood [20] through ABCA1 and ABCG1 [47], but in the injured spinal cord ABCA1 expression is dramatically reduced [8]. This implicates ABCA1 reduction as a driver of foam cell formation. Upon ESC-M treatment, enhanced the expression of both transporters is induced as well as increased lipid exocytosis and resolution of the lipid rich injury epicenter. It could be argued that the less lipid-laden macrophages could be the result of fewer macrophages or decreased myelin phagocytosis. This is unlikely as there was no significant change in F4/80 positive cells between treatments in vivo and ESC-M significantly enhanced myelin phagocytosis and subsequent exocytosis in vitro.

## Conclusion

In conclusion, the present study demonstrates the applicability of embryonic stem cell conditioned media to effectively treat SCI by reducing lipid accumulation, promoting a M2-like state and improving functional recovery. This strategy represents a significant paradigm shift in ESC based therapy, and is one that circumvents the current complications limiting safe clinical application of whole cell transplantation. While additional research is needed to determine the specific factors of the ESC secretome that induce these changes, we can currently conclude that ESC-M is sufficient to promote function recovery in a murine model of SCI.

## Methods

### Mice strains

C57BL/6, B6.129P-Cx3CR1^tm1Litt/J^, and B6.Cg-Tg(CAG-mRFP1)1F1Hadj/J mice were purchased from Jackson Laboratory (Bar Harbor, ME) and maintained in the pathogen-free animal facility in Florida State University College of Medicine. All animal protocols were approved by the Animal Care and Facilities Committee of Florida State University.

### Reagents and antibodies

All chemicals were purchased from Sigma-Aldrich (St. Louis, MO) and cell culture media was purchased from Invitrogen (Carlsbad, CA) unless specially indicated. The F4/80 hybridoma cell line is from American Tissue Culture Collection (ATCC, Manassas, VA, USA). Primary antibodies included in the study are listed in Table 1. Alexa Fluor-conjugated secondary antibodies were purchased from Invitrogen.

**Table 1:** Primary antibodies

Epitope	Catalog Number	Manufacturer
ABCA1	89352-256	Genetex
ABCG1	sc-650	Santa Cruz
Arginase-1	4685	Cell Signaling
GAPDH	2118	Cell Signaling
GFAP	Z0334	Dako
Gr-1	RB6-8C5	eBioscience

### Preparation of mouse bone marrow-derived macrophages

Mouse bone marrow-derived macrophages (BMDMs) were prepared as previously described [17]. Briefly, bone marrow from 6 to 8 weeks old mice was collected from femoral shafts by flushing the marrow cavity with Dulbecco’s modified eagle medium (DMEM) supplemented with 1 % fetal bovine serum (FBS). The cell suspensions were passed through an 18-gage needle to obtain a single cell suspension. Cells were cultured for 7 days in DMEM supplemented with 15 % conditioned medium from L929 cells [a source of macrophage colony-stimulating factor (M-CSF)], 10 % FBS, and 1 % penicillin/streptomycin (Corning).

### Preparation of mouse microglial and astrocyte cultures

Primary astrocyte and microglial cultures were obtained from C57BL/6 mice between 2 and 3 days old. The cerebral cortices were dissected and dissociated prior to plating in tissue culture flasks. The mixed glial cultures were grown in DMEM/F12 supplemented with 10 % FBS, and 1 % penicillin/streptomycin (Corning) until reaching confluence. The flasks were then shaken at 200 rpm for 3 h at 37 °C and microglia in the supernatant were isolated. The cells remaining in the flask after microglia isolation were cultured as an enriched astrocyte primary culture [48].

### ESC culture and preparation of ESC-conditioned medium (ESC-M)

The mouse green fluorescent protein (GFP) expressing-ESC line (F12) derived from a C57BL/6 mouse was kindly donated by Professor Melitta Schachner (Rutgers University). As previously described, freshly thawed ESCs (P0) were seeded into a 100 mm tissue culture dish with mitomycin-treated murine embryo fibroblasts (MEFs) as feeder layer. ESC culture medium was composed of 10^3^ U/ml Leukemia Inhibitory Factor (LIF) (Millipore, CA, USA), 15 % FBS, 1 % non-essential amino acids solution (MEM), 200 mM L-glutamine, 1 % nucleoside solution, 1 % 100nM Na-Pyruvate, 0.2 % 2-β-Mercaptoethanol and DMEM. After 3–4 passages, ESCs were transferred to a 0.1 % gelatin-coated tissue culture dish without a feeder layer, and sub-cultured every 2–3 days prior to collection of the conditioned ESC media (ESC-M). The collected ESC supernatant was spun at 2500 RPM for 10 min and then filtered through a 0.4-μm filter (Corning, USA) to remove any debris. ESC-M was collected in this manner from multiple passages and pooled and stored at −80 °C prior to use. Control culture medium (without ESCs) was used as control medium (CON-M) in this study.

### Preparation of myelin debris

Myelin debris was isolated from the brains of 3 month old mice by sucrose density gradient centrifugation, as described previously [12]. The endotoxin concentration of myelin debris was under the detection limit of the Limulus Amebocyte Lysate assay. Myelin debris was added to cells at a final concentration of 1 mg/mL in all experiments.

### Spinal cord injury

Thoracic spinal cord contusion injuries were performed on 8–10 weeks old female C57BL/6 mice. The laminectomy was performed to expose the spinal cord at T10. The contusion injury was induced using the NYU impactor with a 10 g rod dropped 6.25 mm from the cord surface [49]. Mice contused with asymmetrical injuries were excluded from experimental analysis.

### Treatment groups and basso mouse scale for locomotion score

One mL ESC-M or CON-M was injected intraperitoneally (*i.p.*) per mouse immediately after the surgery and every 3^rd^ day until the mice were sacrificed. Basso Mouse Scale (BMS, 0–9) was applied to assess the locomotor recovery [28] every two days, and the scores were recorded from the 1 day post-injury until sacrificed. As a second measure of locomotor activity, a horizontal ladder beam task was used to measure coordination [30]. To limit bias, all experiments were performed double blinded.

### Histology and histological staining

Mice were transcardially perfused with 0.9 % saline followed by 4 % paraformaldehyde. Segments of spinal cord encompassing the injury site were removed and post-fixed in 4 % paraformaldehyde overnight at 4 °C then cryoprotected in 30 % sucrose overnight at 4 °C. The spinal bones were removed prior to flash freezing in OTC and slicing into 7 μm-thick parasagittal sections with a cryostat microtome. For Immunofluorescence (IF) staining, sections were incubated with 10 mM CuSO_4_ in 50 mM NH_4_Ac (pH 5.0; acidified with acetic acid) for 10 min to reduce autofluorescence after counterstaining with Hoechst [50]. Oil Red O (ORO) staining was applied to visualize lesion lipid accumulation after SCI in vivo and macrophages phagocytosing myelin debris in vitro.

### Western blot

After washing with ice-cold PBS, cells were lysed in RIPA buffer containing a phosphatase inhibitor and proteinase inhibitor cocktail (Amresco). The samples were adjusted to equal protein concentrations and loaded into a sodium dodecyl sulfate-polyacrylamide gel electrophoresis (SDS-PAGE) gel. Proteins on the gel were transferred to PVDF membranes (GE Healthcare, UK) and blocked in 5 % milk or BSA (according to antibody manufacturer’s instructions) in Tris-buffered saline containing 0.1 % Tween 20 (TBST) for 1 h at RT. The membranes were incubated with appropriate primary antibody solution overnight at 4 °C. After rinsing in TBST, the membranes were incubated with appropriate secondary antibody for 1 h. Proteins were visualized by ECL plus western blot detection system (GE Healthcare, UK).

### RNA isolation and quantitative real-time-PCR

Total RNA was isolated by TRIZOL and reverse-transcribed into cDNA by using oligo-dT primers and SuperScript II Reverse Transcriptase. The following primer pairs of cytokines were specifically designed for mRNA: TNF-α (5′-GCCTCTTCTCATTCCTGCTTG-3′ and 5′-CTGATGAGAGGGAGGCCATT-3′) and Arginase-1 (5′- TTGCGAGACGTAGACCCTGG-3′ and 5′- CAAAGCTCAGGTGAATCGGC-3′). The ABI7900HT detection system (Applied Biosystems, UK) was used for quantitative real-time (qRT)-PCR. SYBR Green dye (Applied Biosystems) was used to monitor the replication of PCR products. Quantification of products were obtained by standard curve and normalized to GAPDH (5′- ATCAACGACCCCTTCATTGACC-3′ and 5′- CCAGTAGACTCCACGACATACTCAGC-3′) amount. The gene fold change expression levels were represented by the ratio of target/GAPDH.

### Macrophage apoptotic cell phagocytosis assay

BMDMs were treated with myelin debris for 24 h and non-ingested myelin debris was washed away. Cells were then treated with CON-M or ESC-M for 24 h. For the preparation of apoptotic cells, mouse thymocytes were incubated with 10 μM dexamethasone at 37 °C for 4 h [51]. Apoptotic thymocytes were then stained with a 10 μM solution of carboxyfluorescein diacetate succinimidyl ester (CFSE) to label cells in suspension for 20 min at RT [52], and 5 × 10^5^ cells were added to each well of macrophages in a 96-well cell culture plate. After a 30 min interaction at 37 °C in 5 % CO_2_ atmosphere, the wells were washed in PBS at 4 °C to remove non-ingested apoptotic cells. The proportion of macrophages ingesting at least one thymocyte was counted using inverted light microscopy [8].

### Enzyme-linked immunosorbent assay (ELISA) to detect myelin debris phagocytosis

Since myelin basic protein (MBP) is a major component protein of CNS myelin [53], and is not produced by phagocytes, MBP levels detected in phagocyte cytoplasm are proportional to levels of myelin debris phagocytosed [53, 54]. In this study, ELISA for MBP was applied to study and quantify myelin debris phagocytosis of macrophages [55]. In brief, BMDMs were incubated with myelin debris for indicated time. Non-ingested myelin debris was washed away and intracellular protein was collected by lysis buffer. The protein concentration of cell lysate was determined by Pierce BCA Protein Assay Kit (Thermo Scientific, IL), and 100 μl of coating buffer with same amount protein per well was loaded into 96-well plate to coat antigen at 4 °C overnight. Through coating, blocking, incubating with antibodies and detecting was performed via standard ELISA procedures.

### Analysis of free cholesterol

BMDMs were treated with myelin debris (1 mg/mL) for 24 h at which point undigested myelin was washed away. Cells were then incubated with CON-M or ESC-M for additional 3 h, 6 h, 12 h, or 24 h. The supernatant was collected at the given time points and the free cholesterol in the supernatant was determined by the enzymatic, fluorometric method, using the cholesterol assay kit (Cayman Chemical, MI).

### Statistical analysis

Images were quantified using Image Pro Plus 6 (Media Cybernetics, USA) and positive signal was reported as mean signal density. Results showed in figures were presented as mean ± SD (unless otherwise indicated) with *n* representing the number of biological replicates. Student’s unpaired *t*-test was used to evaluate statistical significance between two groups and ANOVA was used for multiple group comparisons. Chi-square test was used to assess the regained coordination between treatment groups. A p-value less than 0.05 was considered significant.

## Ethics approval

All animal (mouse) work was approved by the Animal Care and Facilities Committee of Florida State University. No human subjects have been involved.

## Statement of consent for publication

Not applicable

## Abbreviations

Arg-1: arginase-1
BMDMs: bone marrow-derived macrophages
BMS: Basso mouse scale
CNS: central nervous system
CON-M: control culture media (cell-free)
ESC-M: ESC-conditioned media (cell-free)
ESCs: embryonic stem cells
MBP: myelin basic protein
Mye-Mϕ: myelin-laden macrophage
Naïve-Mϕ: BMDMs without myelin treatment
ORO: Oil Red O
SCI: spinal cord injury
